# The role of histone post-translational modifications in cancer and cancer immunity: functions, mechanisms and therapeutic implications

**DOI:** 10.3389/fimmu.2024.1495221

**Published:** 2024-11-15

**Authors:** Xiaohong Duan, Zhiyao Xing, Lu Qiao, Shan Qin, Xuejing Zhao, Yanhua Gong, Xueren Li

**Affiliations:** ^1^ School of Disaster and Emergency Medicine, Faculty of Medicine, Tianjin University, Tianjin, China; ^2^ Institute of Disaster and Emergency Medicine, Faculty of Medicine, Tianjin University, Tianjin, China; ^3^ Medical School, Faculty of Medicine, Tianjin University, Tianjin, China; ^4^ Tianjin University and Health-Biotech United Group Joint Laboratory of Innovative Drug Development and Translational Medicine, School of Pharmaceutical Science and Technology, Faculty of Medicine, Tianjin University, Tianjin, China; ^5^ Department of Respiratory Medicine, Jinnan Hospital, Tianjin University, Tianjin, China; ^6^ Department of Respiratory Medicine, Tianjin Jinnan Hospital, Tianjin, China; ^7^ The Province and Ministry Co-sponsored Collaborative Innovation Center for Medical Epigenetics, Key Laboratory of Immune Microenvironment and Disease (Ministry of Education), Department of Biochemistry and Molecular Biology, School of Basic Medical Sciences, Tianjin Medical University, Tianjin, China

**Keywords:** histone PTMs, methylation, acetylation, HMTs, KDMs, HDAC, cancer

## Abstract

Histones play crucial roles in both promoting and repressing gene expression, primarily regulated through post-translational modifications (PTMs) at specific amino acid residues. Histone PTMs, including methylation, acetylation, ubiquitination, phosphorylation, lactylation, butyrylation, and propionylation, act as important epigenetic markers. These modifications influence not only chromatin compaction but also gene expression. Their importance extends to the treatment and prevention of various human diseases, particularly cancer, due to their involvement in key cellular processes. Abnormal histone modifications and the enzymes responsible for these alterations often serve as critical drivers in tumor cell proliferation, invasion, apoptosis, and stemness. This review introduces key histone PTMs and the enzymes responsible for these modifications, examining their impact on tumorigenesis and cancer progression. Furthermore, it explores therapeutic strategies targeting histone PTMs and offers recommendations for identifying new potential therapeutic targets.

## 1 Introduction

The regulation of chromatin structure, nucleosome positioning, and gene transcription is primarily controlled by histone proteins. The nucleosome, the fundamental unit of chromatin, consists of a central histone octamer, around which approximately 1.75 left-handed superhelical turns of DNA are wrapped (1, 2). Each nucleosome is made up of two identical subunits, and each subunit contains four core histones: H2A, H2B, H3, and H4. Additionally, histone H1, which acts as a linker, is not part of the nucleosome itself but plays a crucial role in stabilizing the DNA between nucleosomes (3). These histones undergo various forms of post-translational modifications (PTMs), which serve as epigenetic markers that influence their interaction with DNA. Under normal physiological conditions, these histone PTMs are essential for maintaining nucleosome structure and functioning as regulatory mechanisms. They play vital roles in key cellular processes, including DNA replication, gene expression, DNA damage repair, and chromatin organization (4).

**Figure 1:**
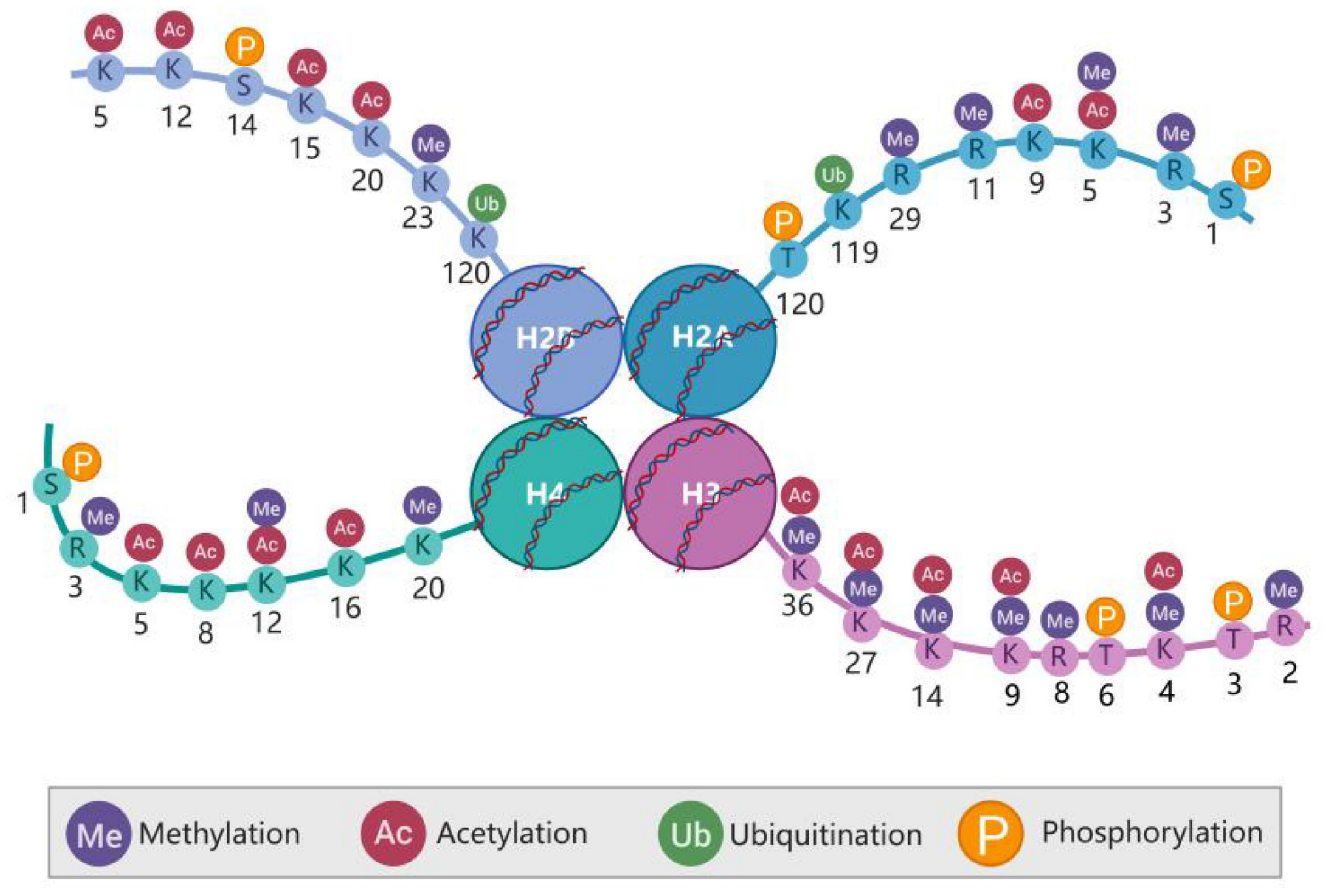
Post-translational modifications (PTMs) of the histone amino terminus. Histones in nucleosomes (two each of H2A. H2B. H3. and H4), Histone tails are subject to various PTMs that affect not only the overall compression of chromatin but also gene expression. Created in BioRender. Xing, Z. (2024) https://BioRender.com/l06b379.

At least eleven types of post-translational modifications (PTMs) have been identified on histones, including methylation, acetylation, propionylation, butyrylation, formylation, ubiquitylation, phosphorylation, sumoylation, citrullination, proline isomerization, and ADP ribosylation, occurring at more than 60 different amino acid residues (5) (**Figure 1**). These modifications can occur in various combinations, contributing to a wide range of biological functions. Upon histone modification, the chromatin structure is altered, which subsequently influences the interaction between histones and DNA, thereby regulating gene transcription.

**Figure 2:**
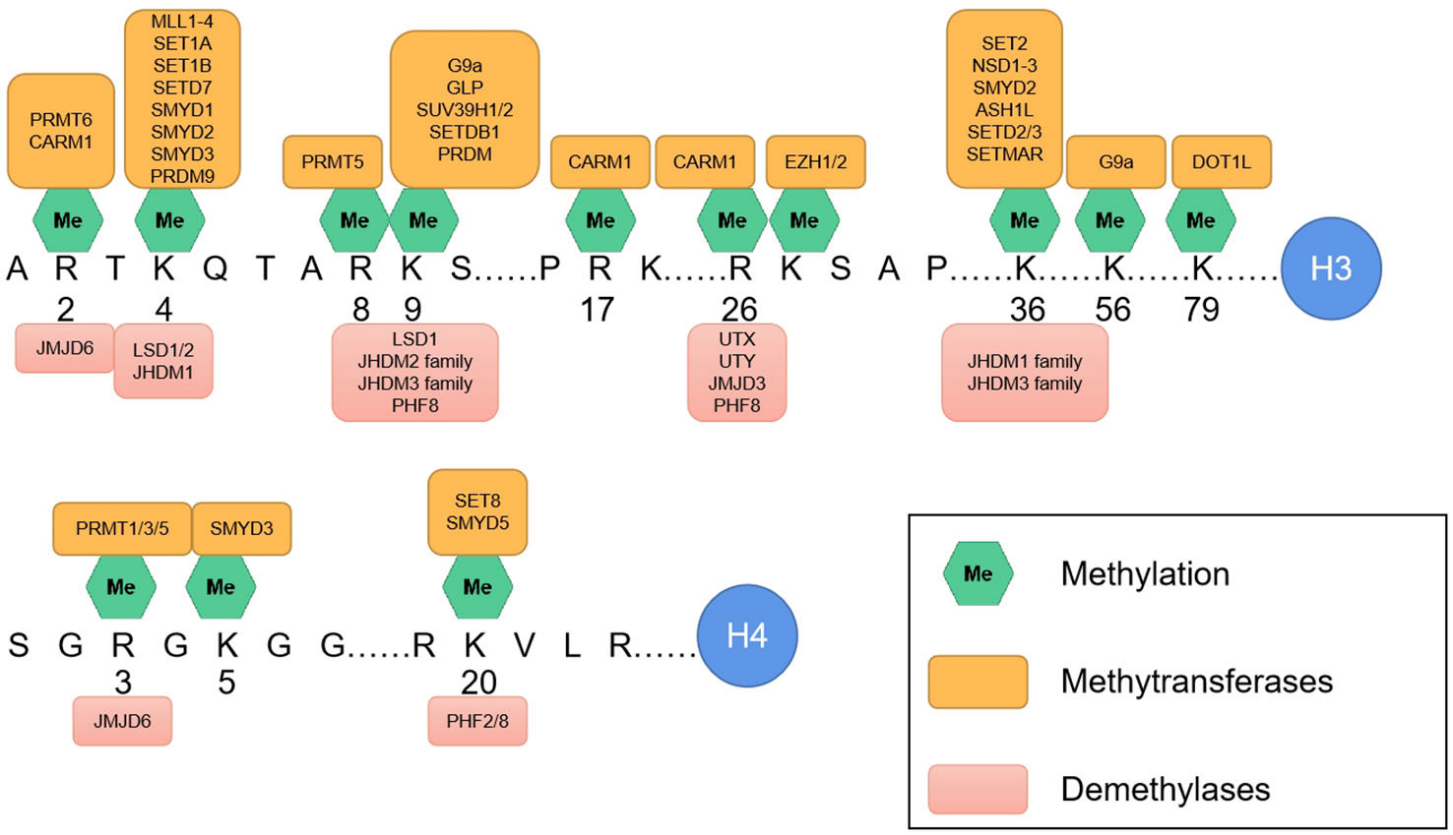
The main methylation sites on the amino termini of H3 and H4. Along with the associated methyltransferases (above) and demethylases (below).

Most histone post-translational modifications (PTMs) are localized within the N-terminal tail domain of core histones, although some crucial PTMs involved in histone–DNA and histone–histone interactions also occur in the globular domain of core histones (6–8). The structural domains at the ends of histone tails are positively charged and interact with negatively charged DNA. Much of the N-terminal part of the histone tail does not participate in nucleosome assembly and protrudes from the core structure, making it more suitable for interactions with the surrounding environment and, thus, more susceptible to PTMs (9). Compared with those of H2A and H2B, the histone tails of H3 and H4 are particularly vulnerable to PTMs. PTMs on histone tails are typically recognized by “reader” or effector proteins, which, in turn, regulate chromatin function (**Figure 2**). Histone methylation and acetylation predominantly occur on the N-terminal tail and are key regulators of gene transcription. PTMs in the globular domain of histones can disrupt histone–histone interactions, destabilize nucleosomes or alter histone-DNA interactions, impacting nucleosome dynamics and chromatin function, often without the need for effector proteins.

Mounting evidence indicates that histone post-translational modifications (PTMs) play essential roles in a variety of biological processes, including cell differentiation and organismal development. The dysregulation of histone PTMs under pathological conditions is closely associated with the onset and progression of major human diseases, especially cancer. Enzymes such as histone methyltransferases, demethylases, acetyltransferases, and deacetylases regulate gene expression through these modifications. For instance, histone methylation typically results in gene silencing, while acetylation activates gene transcription; in contrast, demethylation and deacetylation generally reverse these effects. The abnormal expression of these modifying enzymes is a key factor contributing to tumor development and progression. Thus, understanding the biological roles of histone PTMs is critical for elucidating their pathophysiology. This review emphasizes the role of histone PTMs in cancer and explores the mechanisms underlying abnormal modification events. Additionally, this review discusses the potential of developing therapeutic drugs that target histone-modifying enzymes, offering new directions for identifying novel therapeutic targets and strategies for cancer treatment.

## 2 Histone modifications related to cancer

### 2.1 Histone methylation

#### 2.1.1 Histone methylation in cancer

Histone methylation predominantly occurs on lysine and arginine residues and represents a critical post-translational modification catalyzed by histone methyltransferase enzymes. This modification can either activate or repress transcription, depending on the specific sites involved. For example, methylation at H3K4, H3K36, and H3K79 is generally linked to transcriptional activation, whereas methylation at H3K9, H3K27, and H4K20 is typically associated with transcriptional repression (10). The impact of methylation also varies on the basis of degree of methylation. For example, monomethylation of H4K20 (H4K20me1) is observed in active gene bodies, whereas trimethylation of H4K20 (H4K20me3) is associated with gene repression and chromatin compaction (11, 12). Additionally, the position of the methylated lysine residues relative to the DNA sequence plays a crucial role in gene regulation. For example, H3K9me3 at the promoter region is linked to gene silencing, whereas H3K9me3 within the gene body is often found in inducible genes. Since this modification is electrically neutral and chemically inert, it relies on other proteins with binding motifs to exert its function. Proper histone methylation is essential for genomic programming during development. However, during tumorigenesis, dysregulated histone methylation promotes tumor cell proliferation, migration, and invasion, ultimately contributing to tumor progression and poor prognosis (**Table 1**).

**Table 1:** Abnormal histone methylation in cancer.

Cancer type	Relevant histone modifications	Abnormal expression pattern	Ref
Acute Myeloid Leukemia	H3K9me3	Downregulation	Laura Monaghan (13)
Gastric cancer	H3K9me3	Upregulation	Park YS (14)
Colorectal cancer	H3K9me3	Downregulation	Benard A (15)
Glioblastoma	H3K27me3	Downregulation	Pathak P (16)
Glioblastoma	H3K4me3	Downregulation	Pathak P (16)
Breast cancer	H3K4me3	Upregulation	Luisa Berger (17)
Hepatocellular carcinoma	H3K4me3	Upregulation	Gao SB (18)
Hepatocellular carcinoma	H3K27me3	Upregulation	Duan JL (19)
Pancreatic Adenocarcinoma	H3K4me2	Downregulation	Ananya Manuyakorn (20)
Lung cancer	H3K4me2	Downregulation	Barlési F (21)

##### 2.1.1.1 H3K9me3

The expression levels of numerous histone modification markers are closely linked to the prognosis of various human cancers. Among these, H3K9me3 is a key histone modification marker that plays a significant role in tumor development and patient outcomes. H3K9me3 is generally associated with gene transcriptional silencing and influences cancer progression in multiple ways. On the one hand, H3K9me3 contributes to the abnormal silencing of tumor suppressor genes, thereby promoting tumor progression and leading to poorer patient prognosis. For instance, in HCT116 cells, the promoter and adjacent 3’ regions of the tumor suppressor gene DCC are enriched with the repressive H3K9me3 marker, which inhibits DCC transcription and promotes colorectal cancer development (22). Elevated H3K9me3 levels are also prognostic markers in cancers such as acute myeloid leukemia, gastric adenocarcinoma, salivary carcinoma, and bladder cancer (23–26). On the other hand, H3K9me3 helps to repress the aberrant expression of oncogenes and regulates the silencing of repetitive sequences in the genome. Studies have shown that higher H3K9me3 immunostaining scores are inversely correlated with disease recurrence, particularly distant metastasis, and improved disease-free survival in patients with non-small cell lung cancer (27). Furthermore, reduced levels of both H3K9me3 and H4K20me3 are associated with shorter survival times and increased tumor recurrence rates in patients with early-stage colon cancer (15).

##### 2.1.1.2 H3K4me3

H3 lysine 4 (H3K4) methylation is among the most extensively studied histone modifications due to its strong association with gene expression and cancer development. This methylation is catalyzed by the SET1/COMPASS complex, which comprises several lysine methyltransferases and essential subunits, including six catalytic members: SETD1A, SETD1B, and MLL1-4 (28). H3K4me3 is typically found at transcription start sites (TSSs) and is believed to enhance transcription by recruiting PHD finger-containing proteins, such as TATA-box binding protein-associated factor 3 (TAF3), which play critical roles in transcription initiation (29). Additionally, H3K4me3 at promoter regions can counterbalance repressive histone modifications such as H3K9me3 and H3K27me3, helping to activate gene transcription (30–32). H3K4me3 is a hallmark of actively transcribed genes and has been implicated in promoting changes in gene expression and advancing tumor progression. Recent research has indicated that H3K4me3 actively participates in driving the progression of several cancers, including lung cancer, liver cancer, multiple myeloma, and prostate cancer (33–37). Notably, in gastric cancer (GC) patients, H3K4me3 is significantly upregulated at the TM4SF1-AS1 locus, promoting the expression of TM4SF1-AS1, which in turn inhibits apoptosis in gastric cancer cells (38). While most expressed genes present H3K4me3 restricted to the promoter and 5’ regions of the gene body, a subset of genes exhibit broader H3K4me3 regions (39). These broad domains often identify genes involved in crucial functions such as cell identity, tumor suppression, and disease-related processes (32, 40). For example, highly metastatic triple-negative breast cancer cells show higher expression levels of oncogenes linked to broad H3K4me3 domains compared to normal breast epithelial cells and less malignant breast cancer cell lines (39).

##### 2.1.1.3 H3K27me3

H3K27 methylation is catalyzed by polycomb repressive complex 2 (PRC2), a key regulator whose subunits can recognize the H3 tail for complex binding (41). The primary function of PRC2 is to deposit methyl groups onto the lysine 27 residue of histone H3 (H3K27), resulting in gene repression (42). This trimethylation of H3K27 is mediated by the histone methyltransferase enhancer zeste homolog 2 (EZH2), an essential component of PRC2 (43). H3K27me3 plays a critical role in cell differentiation, and studies have demonstrated that the disruption of PRC2 impairs the differentiation of embryonic stem cells (44).

In addition to its pivotal role in development, alterations in H3K27me3 are observed in various cancer types, where H3K27me3 contributes to different stages of tumor initiation, progression, and metastasis. Both the upregulation and downregulation of H3K27me3 have been implicated in numerous cancers. For example, in ovarian cancer, overexpression of EZH2 leads to increased H3K27me3 levels, which suppresses the expression of genes such as E-cadherin, TIMP2, and TIMP3, all of which are involved in cell migration. Their repression facilitates tumor metastasis (45). EZH2 also modulates the expression of DAB2IP by trimethylating H3K27 at the DAB2IP promoter. DAB2IP, known for its Ras-GTPase activity, suppresses cancer stem cell phenotype in several cancers, and its repression by EZH2 promotes cancer cell stemness (46, 47).

Conversely, the loss of H3K27me3 can also contribute to tumor development. In such cases, reduced H3K27me3 may lead to the activation of tumor suppressor genes, accelerating tumor progression. In diffuse midline gliomas, for example, the loss of H3K27 trimethylation is a primary driver of tumor growth (48). This reduction in H3K27 methylation promotes glial cell stemness and silences tumor suppressor genes (42). Thus, dynamic changes in H3K27me3 play multifaceted and complex roles at various stages of tumor initiation and progression.

#### 2.1.2 Histone methyltransferases in cancer

Histone methyltransferases (HMTs) are categorized into two main subfamilies: histone lysine methyltransferases (HKMs) and histone arginine methyltransferases (HRMs) (49). Lysine methyltransferases (KMTs) can be further divided into two families based on the sequence of their catalytic domain: those with the SET domain (located in the histone tails) and those with non-SET domains (located in the histone core) (50–52). All HKMTs, except those in the Dot1 family, contain a conserved enzymatic SET [SU(VAR)3-9, E(Z), and TRX] domain, which was initially discovered in su(var)3-9, enhancer-of-zeste, and trithorax proteins (52). The SET domain includes enzymes such as SUV39H1/2, G9a, EZH2, GLP, and SETDB1 (53).

In the histone arginine methyltransferase (HRM) subfamily, protein arginine methyltransferases (PRMTs) methylate arginine residues in histones through mono-, symmetric-, or asymmetric-dimethylation (54). Currently, nine PRMTs have been identified in mammals, all of which possess four conserved motifs. Mammalian PRMTs are classified into two groups on the basis of the type and position of methylation. Class I includes PRMT1, 3, 4, 6, and 8, which catalyze monomethylation and asymmetric dimethylation of arginine. Class II comprises PRMT5 and PRMT7, which catalyze monomethylation and symmetric dimethylation of arginine (55–57).

The dysregulated expression of histone methyltransferases (HMTs) can result in aberrant histone methylation of cancer-related genes, thereby contributing to tumor development. The histone-lysine N-methyltransferase (KMT2) family plays crucial roles in regulating transcription. Mutations in the KMT2 family are among the most frequently observed genetic aberrations in various cancers, including hematological malignancies and solid tumors such as colorectal, lung, endometrial, breast, bladder, and brain cancers (58–63). Members of the PRMT family have also gained importance in the study of different cancer types. The overexpression and dysregulation of PRMT4, PRMT5, and PRMT7 are known to drive the progression of several hematological malignancies and solid tumors, making them effective therapeutic targets (64–66). Therefore, PRMT family members can serve as effective targets for cancer therapy.

Aberrant expression of HMTs disrupts the transcriptional regulation of genes linked to disease, including oncogenes and tumor suppressor genes, which can promote tumorigenesis. HMTs are associated with chemotherapy resistance and immune evasion, highlighting their potential as therapeutic targets. Chemically targeting these enzymes represents a promising approach for the development of novel cancer therapies.

##### 2.1.2.1 SETD1A

SETD1A is a histone lysine methyltransferase that contains the SET domain and is part of the SET1/COMPASS complex family, alongside its paralog SETD1B (28, 67). SETD1A specifically methylates H3K4, a modification crucial for the transcriptional activation of genes that regulate the self-renewal and differentiation of embryonic stem cells (68). Additionally, SETD1A plays a significant role in maintaining mitosis and cell proliferation. Research has shown that SETD1A is upregulated in various cancers, and its overexpression is linked to accelerated tumor cell proliferation and invasion, which is often correlated with poor prognosis.

In lung cancer, high levels of SETD1A expression drive the deposition of H3K4me3 on the promoters of key oncogenes such as MYC, GLI1, FOXM1, and DNMT1. This promotes the transcription of these oncogenes, thereby facilitating the onset and progression of lung cancer (33). Furthermore, studies indicate that SETD1A contributes to the development of gastric cancer by increasing H3K4me3 levels at hypoxia response elements in the promoters of HK2 and PFK2. This, in turn, enhances the transactivation of HIF1α and upregulates the expression of its target genes, ultimately leading to increased glycolysis in gastric cancer cells (69). Similarly, SETD1A is overexpressed in pancreatic ductal adenocarcinoma (PDAC) and is associated with poor patient prognosis. It binds to the promoter of the oncogenic protein ATP-dependent DNA helicase gene RUVBL1, increasing H3K4me3 levels and promoting transcriptional regulation of the gene, which plays a pivotal role in PDAC cell proliferation and motility (70). These findings suggest that SETD1A may serve as a potential predictive marker for various cancer types.

##### 2.1.2.2 KMT2D

Lysine-specific methyltransferase (KMT2D), also known as myeloid/lymphoid or mixed-lineage leukemia 2 (MLL2), is a key histone methyltransferase essential for regulating gene transcription. It specifically targets histone H3 lysine 4 (H3K4), whose methylation serves as a marker for gene activation. KMT2D, along with SETD1A, is a member of the SET/MLL (mixed lineage leukemia) methyltransferase family, which is conserved across species, from yeast to mammals. In humans, this family includes six H3K4 methyltransferases (HMTs): MLL1 (MLL/KMT2A), MLL2 (KMT2B), MLL3 (KMT2C), MLL4 (KMT2D), SETD1A (KMT2F), and SETD1B (KMT2G) (58, 71).

In recent years, KMT2D has emerged as one of the most frequently mutated genes in various cancers and human diseases, including lymphoma, medulloblastoma, and gastric cancer (72–74). Mutations in KMT2D often result in a loss of function, suggesting its role as a tumor suppressor in various tissues. The absence of KMT2D affects the proliferation and migration of colorectal cancer cell lines. KMT2D regulates H3K4 monomethylation and is associated with enhancer elements in the HCT116 cell line. The SET domain of the enzyme is critical for maintaining effective H3K4 monomethylation, and its activity is directly involved in regulating H3K4me1, which is essential for sustaining tumor cell proliferation.

Moreover, KMT2D plays a role in addressing tumor resistance. For example, drugs targeting the PI3K signaling pathway are effective in some breast cancer patients; however, estrogen receptor (ER)-positive breast cancer patients often develop resistance to these therapies. Toska et al. reported that PI3K inhibition activates KMT2D and that H3K4 methylation catalyzed by KMT2D leads to a more open chromatin state, facilitating estrogen receptor-dependent transcription (75). As a result, researchers have suggested that combination therapy consisting of PI3K inhibitors and KMT2D inhibitors may be more effective than PI3K inhibitors alone.

##### 2.1.2.3 PRMT1

PRMT1 is the predominant type 1 protein arginine methyltransferase (PRMT), accounting for more than 85% of arginine methylation in mammals, with histone H4 as its primary methylation target. Arginine dimethylation of histone H4 (H4R3me2a) enhances histone acetylation, chromatin accessibility, and transcriptional activation. Recent studies have underscored the critical role of arginine methylation in various human diseases, particularly cancer. Elevated levels of PRMT1 are linked to poor prognosis in many cancer types.

Research conducted by Ku et al. demonstrated that high PRMT1 levels are associated with unfavorable outcomes in both human and mouse pancreatic cancer patients (76). PRMT1 activity is essential for regulating chromatin accessibility and controlling the expression of key glycolytic genes, such as GLUT1 and HK2. Furthermore, inhibiting PRMT1 can disrupt KRAS-driven glycolysis in pancreatic ductal adenocarcinoma (PDAC), thereby affecting tumor metabolism.

PRMT1 also acts in synergy with SMARCA4, an ATPase subunit of the SWI/SNF chromatin remodeling complex, to drive the progression of colorectal cancer (CRC) (77). Mechanistically, it was shown that H4R3me2a directly recruits SMARCA4, enhancing the proliferative, colony-forming, and migratory capacities of CRC cells by activating EGFR signaling pathways. PRMT1 is further involved in numerous interactions with transcription factors and gene promoters. The overexpression and aberrant splicing of PRMT1 are directly implicated in the development of several cancers, including breast, lung, and bladder cancers and leukemia (78–81).

#### 2.1.3 Histone demethylases in cancer

Histone methylation is a reversible modification that is dynamically regulated by the interplay between histone methyltransferases and histone demethylases. Histone demethylation is catalyzed by histone demethylases (HDMs), which remove methyl groups from specific amino acids on the N-terminal tails of histones. These enzymes primarily target lysine (K) residues on histone H3, such as K4, K9, K27, and K36, and are also known as lysine demethylases (KDMs). Currently, two evolutionarily conserved families of histone demethylases have been identified: lysine-specific demethylases (LSDs) and demethylases containing the Jumonji C (JmjC) domain (JHDM). These two families utilize different mechanisms to remove methyl groups (82, 83).

The LSD family includes LSD1 and LSD2, which demethylate mono- and dimethylated lysine residues through a flavin adenine dinucleotide (FAD)-dependent amine oxidase reaction. LSD1, also known as KDM1A or AOF2, was the first discovered histone lysine demethylase. It removes mono- and dimethyl groups from lysine 4 (H3K4me1/2) or lysine 9 (H3K9me1/2) of histone H3, serving as either repressors or activators of gene expression (84). However, owing to its reliance on FAD and protonated nitrogen, LSD1 can only demethylate mono- or dimethylated lysines and is ineffective against trimethylated lysines (82, 85). The catalytic mechanism of LSD1 limits its ability to demethylate trimethylated lysine, a widely observed modification. This has led researchers to propose that other catalytic mechanisms may exist for histone lysine demethylation.

Zhang et al. discovered that F-box and leucine-rich repeat protein 11 (FBX11) possesses histone demethylase activity and contains a JmjC domain, classifying it as JHDM1A (JmjC domain-containing histone demethylase 1A) (86). JmjC domain-containing proteins exhibit hydroxylase activity, which enables their demethylation function. JHDM1A specifically removes dimethylation marks from H3K36me2 on histone H3 in the presence of divalent iron ions and α-ketoglutarate. The JHDM1A protein comprises several domains: a JmjC domain, an F-box domain, a PHD, a zinc finger domain, and three leucine-rich repeat regions, with the JmjC domain serving as the catalytic domain. On the basis of sequence information, proteins containing the JmjC domain are classified into seven families: JHDM1, JHDM2, JHDM3, JARID1, UTX/UTY, PHF8, and those containing only the JmjC domain. The JmjC family has 30 members, nearly 20 of which have demonstrated histone demethylase activity. Unlike LSDs, JmjC domain-containing proteins do not require a hydrogen donor, allowing them to demethylate all three methylation states of lysine residues.

Proteomic analyses have shown that histone arginine methylation is also a reversible modification. However, the “arginine demethylases” (RDMs), which directly remove methyl groups from arginine, remain poorly characterized. The most studied candidates are peptidylarginine deiminase 4 (PADI4) and Jumonji domain-containing protein 6 (Jmjd6) (87, 88). PADIs are Ca2+-dependent enzymes that catalyze the conversion of arginine to citrulline in proteins. They can also convert monomethylated arginine (MMA) to citrulline, affecting histone H3 and H4 modifications. Additionally, Chang et al. identified Jmjd6 as a histone arginine demethylase that specifically demethylates histone H3 at arginine 2 (H3R2) and histone H4 at arginine 3 (H4R3).

These histone demethylases can target both histone and non-histone substrates and are involved in various biological processes, including development and metabolic diseases such as diabetes and cancer. Abnormal expression of histone demethylases is linked to tumorigenesis, cancer progression, and drug resistance (**Table 2**). For example, LSD1 is abnormally expressed in several cancers and has been shown to inhibit cancer cell differentiation while promoting proliferation, metastasis, and invasion. LSD1 overexpression is associated with poor prognosis in conditions such as non-small cell lung cancer, neuroblastoma, pancreatic cancer, prostate cancer, and breast cancer (101–105). Inhibiting LSD1 activity may help reduce or halt the growth of these tumors.

**Table 2:** The mechanism of KDMs in cancer.

KDMs type	Cancer type	Function
KDM1A	NSCLC	Upregulating E-cadherin and promoting epithelial-mesenchymal transition (EMT) (89).
	Glioblastoma	Activating the unfolded protein response pathway (90).
KDM2A	HCC	Upregulating the stemness of liver cancer tumor-initiating cells and promoting sorafenib resistance (91).
KDM3A	Ovarian cancer	Demethylating p53 protein (92).
KDM4B	Prostate cancer	Recruiting AR to the c-Myc (MYC) gene enhancer and increasing c-Myc’s transcription (93).
KDM5A	Ovarian cancer	Decreasing E-cadherin expression and increasing N-cadherin/P-glycoprotein expression (94).
	Lung adenocarcinoma	Decreasing expression of E-cadherin and increasing N-cadherin expression (95).
	Breast cancer	Modulating ER signaling (96).
KDM5B	NSCLC	Promote the growth of cancer stem cells (97).
	Hepatocellular carcinoma	Downregulating PTEN and activating the PI3K/Akt pathway (98).
KDM6A	Osteosarcoma	Inactivating RAF/ERK/MAPK cascades (99).
KDM6B	Breast cancer	Counteracting EZH2-mediated suppression of IGFBP5 (100).

##### 2.1.3.1 JMJD1A

JMJD1A, also known as lysine demethylase 3A (KDM3A), is a member of the Jumonji C (JmjC) family of histone demethylases. JMJD1A plays a key role in promoting the expression and activity of various transcription factors through the demethylation of H3K9, thereby regulating critical biological processes such as spermatogenesis, stem cell activity, and sex determination. Notably, JMJD1A is upregulated in multiple malignant tumors, including neuroblastoma, breast cancer, cervical cancer, non-small cell lung cancer, liver cancer, and gastric cancer (106–111).

In prostate cancer, JMJD1A plays several important roles (112). First, the interaction between JMJD1A and the androgen receptor (AR) promotes AR chromatin binding by demethylating H3K9 on AR target genes. Additionally, JMJD1A regulates the oncogene c-Myc through three distinct mechanisms: (1) JMJD1A induces H3K9 demethylation at the c-Myc enhancer, thereby promoting c-Myc transcription (113); (2) JMJD1A acts as a coactivator of c-Myc by interacting with it and enhancing c-Myc recruitment to chromatin via H3K9 demethylation; and (3) JMJD1A interacts with the protein HUWE1, which is involved in the ubiquitination and degradation of c-Myc (114). By inhibiting c-Myc degradation, JMJD1A enhances the stability of c-Myc.

Furthermore, JMJD1A has been implicated in promoting the progression of urinary bladder cancer (115). It does so by enhancing glycolysis through the coactivation of HIF-1α, contributing to cancer cell growth and survival. These findings highlight JMJD1A as a potential therapeutic target in various cancers.

##### 2.1.3.2 UTX/KDM6A

Lysine-specific demethylase 6A (UTX), encoded by the *KDM6A* gene, is a key component of the COMPASS complex, which plays a critical role in gene activation. UTX functions as part of a transcriptional activation complex that includes MLL2/MLL3 (H3K4 methyltransferases) and P300/CBP histone acetyltransferases (116). This collaborative mechanism enables the removal of the repressive histone mark H3K27me3 and the deposition of activation-associated marks such as H3K27 acetylation and H3K4 methylation, facilitating transcriptional activation.

Mutations in *KDM6A* are frequently observed in various cancer types, particularly in primary multiple myeloma (MM) and certain types of T-cell leukemia (117–119). Loss of UTX in MM promotes tumor cell proliferation, clonogenicity, and adhesion. Moreover, UTX-mutant MM cells exhibit increased sensitivity to EZH2 inhibition both *in vitro* and *in vivo*, which correlates with reduced levels of IRF4 and c-MYC, as well as the activation of IRF4 repressors specific to germinal center B cells, such as BCL6 and IRF1.

Interestingly, UTX can either suppress or promote cancer development through interactions with transcription factors. For example, in breast cancer, UTX regulates the oncogenic functions of estrogen receptor α (ERα) (120). In bladder cancer, UTX functions as a tumor suppressor by localizing to enhancers and regulating key genes involved in bladder differentiation through a catalytic-independent mechanism (121). UTX also attenuates the transcriptional and phenotypic effects of aberrant fibroblast growth factor receptor 3 (FGFR3) activation. These findings suggest that the concurrent loss of UTX function and FGFR3 mutations synergistically drive tumorigenesis in bladder cancer. UTX/KDM6A is encoded on the X chromosome and escapes X inactivation in females. In females, UTX lesions are often homozygous, whereas in males, these mutations are frequently accompanied by the loss of the paralog UTY, further supporting its role as a tumor suppressor.

##### 2.1.3.3 JMJD2

The JMJD2A-D proteins, now commonly referred to as KDM4A-D (lysine demethylases 4 A-D), belong to one of the largest subfamilies of JMJD proteins. These enzymes have garnered significant attention for their ability to recognize and demethylate dimethylated and trimethylated histones, specifically H3K9 and H3K36, as well as trimethylated H1.4K26. The KDM4 family consists of three ∼130-kDa proteins (KDM4A-C) and a smaller member, KDM4D/JMJD2D, which lacks the double PHD and Tudor domains found in the other KDM4 proteins (122). These domains function as epigenome readers, and their absence in KDM4D results in different substrate specificities than those of the other family members.

Various studies have shown that KDM4A/JMJD2A, KDM4B/JMJD2B, and/or KDM4C/JMJD2C are overexpressed in breast cancer, colorectal cancer, lung cancer, prostate cancer, and other tumors and are essential for the efficient growth of cancer cells (122, 123). KDM4A/JMJD2A is the most extensively studied member of the KDM4 family, and it can demethylate H3K9 and H3K36, thereby regulating gene transcription. In breast and prostate cancers, KDM4A forms a complex with estrogen and androgen receptors and activates downstream target genes. Therefore, depletion of KDM4A in ER-positive T47D breast cancer cells reduces the expression of ER target genes (such as the oncogenes c-Jun and Cyclin D1) and leads to decreased cell growth (124). Like in breast cancer cells, the knockdown of KDM4A in multiple colorectal cancer cell lines led to reduced cell proliferation, further increased apoptosis, and delayed G2−M phase progression of the cell cycle (125).

KDM4B and KDM4C, which share structural similarities with KDM4A, exhibit the same target specificity and similar enzymatic activities *in vitro* (126, 127). The expression of KDM4B and KDM4C is elevated in breast tumors. Functionally, KDM4C overexpression has been linked to the development of ER-negative breast cancers, while KDM4B contributes to the tumorigenic transformation of ER-positive breast cancer cells (128–131). Furthermore, KDM4B overexpression has been observed in gastric, bladder, lung, and colorectal cancers, where it is essential for the proliferation, colony formation, invasion, and survival of cancer cells (132, 133).

KDM4D, which is distinct from other KDM4 proteins due to its lack of PHD and Tudor domains, also acts as a coactivator of the androgen receptor, similar to KDM4A and KDM4C, thereby promoting tumor progression. Additionally, KDM4D plays a role in mediating inflammatory responses triggered by cytokines such as TNF-α, potentially influencing tumorigenesis within the tumor microenvironment and immune cells (134).

##### 2.1.3.4 JMJD3/KDM6B

Lysine-specific demethylase 6B (KDM6B, also known as JMJD3) is a member of the UTX/UTY JmjC domain protein subfamily that demethylates H3K27 residues, including both the trimethylated and dimethylated forms, in conjunction with UTX (135). KDM6B promotes gene transcription by removing H3K27me3 marks from the promoters of its target genes, thereby preventing the binding of polycomb repressive complex 2 (PRC2), or by recruiting coactivators and mediating their interactions with transcription factors (TFs), which can be dependent on or independent of its demethylase activity (136, 137). KDM6B is a crucial histone demethylase involved in various biological and pathological processes, including development, inflammation, aging, and cancer.

KDM6B functions as both a tumor suppressor and an oncogene, depending on the cellular context. In certain cancer types, such as neuroblastoma, hepatocellular carcinoma, lung adenocarcinoma, and endometrial cancer, KDM6B functions as a tumor suppressor, and its expression is associated with improved survival rates and prognoses (138–141). For example, in non-small cell lung cancer (NSCLC), KDM6B expression is reduced, and when KDM6B is overexpressed, it restricts cell proliferation and migration while inducing apoptosis. This is achieved by inhibiting the phosphorylation of FOXO1, leading to its accumulation in the nucleus (142). In acute myeloid leukemia (AML), particularly the M2 and M3 subtypes, KDM6B overexpression reduces the number of leukemia stem cells, promotes bone marrow differentiation, and induces cellular senescence by upregulating the expression of C/EBPβ and its target genes (143).

Despite its tumor-suppressive functions, KDM6B can also act as an oncoprotein in certain contexts, promoting tumor progression. In prostate cancer cells, KDM6B is highly expressed, with levels increasing as the disease progresses, particularly in metastatic cases. Additionally, in estrogen receptor α (ERα)-dependent breast cancer, KDM6B forms a complex with ERα and binds to ERα sites in the BCL2 enhancer region, leading to BCL2 overexpression following estrogen treatment (144). KDM6B also induces the expression of SNAI1, promoting epithelial-mesenchymal transition (EMT) and contributing to metastasis in patients (145). In esophageal squamous cell carcinoma (ESCC), the RAS/MEK pathway induces KDM6B overexpression, which is associated with disease stage and patient survival (146). Thus, KDM6B plays dual roles in cancer, acting as either a tumor suppressor or an oncogene, depending on the specific cancer type and context.

### 2.2 Histone acetylation

Acetylation, one of the earliest discovered histone modifications affecting transcriptional regulation, introduces negative charges to lysine residues located in the N-terminal histone tails protruding from the nucleosome (147, 148). These negative charges repel negatively charged DNA, resulting in a more relaxed chromatin structure that allows transcription factors to bind more easily, thereby significantly increasing gene expression (149). The dynamic balance of histone acetylation is regulated by two classes of enzymes: histone deacetylases (HDACs) and histone acetyltransferases (HATs) (150). HDACs can remove acetyl groups from histones, leading to a more compact chromatin structure and the suppression of gene transcription. In contrast, HATs add acetyl groups to histones, resulting in a more relaxed chromatin structure and promoting gene transcription. The dynamic balance between these two classes of enzymes determines the level of histone acetylation at specific gene loci, thereby influencing the expression of the corresponding genes (151).

Histone acetylation plays a vital role in regulating various cellular processes, such as the cell cycle, cell proliferation, apoptosis, differentiation, DNA replication and repair, nuclear transport, and neuronal inhibition (152). Imbalances in histone acetylation are closely associated with tumor development and cancer progression, as aberrations in acetylation can disrupt the regulation of gene expression involved in these key processes.

In various cancers, abnormal changes in H3K27ac modification are often linked to the dysregulation of tumor-related genes, affecting critical cellular processes such as proliferation, differentiation, and apoptosis. Several studies have highlighted the potential of H3K27ac modification changes as biomarkers for cancer diagnosis and prognosis, aiding in identifying the onset and progression of malignancies. In pancreatic ductal adenocarcinoma (PDAC), a highly invasive tumor, the pro-apoptotic protein NOXA serves as a marker of the invasive subtype. Research has demonstrated that inhibition of the transcription factor RUNX1 leads to enrichment of H3K27ac, which, in turn, activates the proximal NOXA promoter region (153). This drug-induced enrichment of H3K27ac triggers NOXA-dependent cell death. Similarly, in cervical cancer cells, the long non-coding RNA EGFR-AS1 promotes migration and invasion while inhibiting apoptosis. Studies have shown that CBP interacts with the promoter of EGFR-AS1, activating H3K27ac and subsequently leading to the upregulation of EGFR-AS1, which influences the WNT pathway through ACTN4, promoting cervical cancer cell growth (153).

In summary, H3K27ac plays a crucial role in cancer development and progression. Given its significance as an epigenetic marker, targeting the mechanisms that regulate H3K27ac, such as CBP/p300, has emerged as a promising therapeutic strategy for treating various cancers.

#### 2.2.1 Histone deacetylases in cancer

Lysine acetylation on histone tails is a highly dynamic process crucial for regulating chromatin structure, transcription, and DNA repair. This process is controlled by two enzyme families: histone acetyltransferases (HATs) and histone deacetylases (HDACs) (154). HATs catalyze the transfer of an acetyl group from acetyl-CoA to the ϵ-amino group of lysine residues on histones, leading to a more relaxed chromatin structure that promotes transcription. In contrast, HDACs remove the acetyl group from histones, resulting in a more compact chromatin conformation that reduces transcription factor accessibility. This dynamic regulation of histone acetylation plays a key role in gene expression and chromatin organization.

HDACs, in particular, serve as critical transcriptional corepressors in a variety of physiological and pathological systems. In mammals, there are 18 HDACs, which are classified into four major classes (155, 156). Class I HDACs (HDACs 1, 2, 3, and 8) are ubiquitously expressed in human cells and are primarily localized in the nucleus. Class II HDACs (HDACs 4, 5, 6, 7, 9, and 10) exhibit tissue-specific expression and shuttle between the nucleus and cytoplasm. Compared with other HDAC classes, class III HDACs, also known as sirtuins (SIRT1–7), are NAD+-dependent and have a unique catalytic mechanism. Finally, Class IV contains only one member, HDAC11, which has been found to deacetylate various histone sites, resulting in low substrate specificity and functional redundancy in certain contexts.

Mutations and abnormal expression of HDACs are frequently observed in human diseases, especially cancer, where the dysregulation of histone acetylation contributes to oncogenesis. As a result, HDACs have emerged as significant therapeutic targets in cancer, with their inhibition leading to increased histone acetylation and potential restoration of normal gene expression patterns in tumor cells. Consequently, the overall pattern of histone acetylation becomes dysregulated in cancer, further driving disease progression.

##### 2.2.1.1 Class I HDACs

All members of the class I subfamily of HDACs are dysregulated in many types of cancer. The overexpression of HDAC1 has been observed in patients with breast cancer, prostate cancer, gastric cancer, and pancreatic cancer, and its upregulation is correlated with poor prognosis (157–160). Specifically, HDAC1 is highly expressed in glioblastoma (GBM) tissues, where it promotes the invasion and migration of GBM cells by regulating the epithelial-mesenchymal transition (EMT) process (161). Moreover, HDAC1 upregulation has been identified as a key factor in the development of drug resistance in ovarian cancer. Enhancing c-Myc-dependent miR-34a expression to target HDAC1 may offer a promising strategy to improve the efficacy of cisplatin treatment in these patients (162).

In human lung cancer cell lines, HDAC2 inactivation leads to apoptosis via the activation of p53 and Bax (163). Conversely, in colorectal cancer cells, the loss or knockdown of HDAC2 induces EMT and lung metastasis by upregulating the long non-coding RNA H19 (lncRNA H19) (164). Additionally, frequent mutations in the CREBBP gene in B-cell lymphomas drive tumorigenesis *in vivo* through the involvement of HDAC3 (165). HDAC3 also plays a crucial role in the development of non-small cell lung cancer (NSCLC) driven by the KL and KP genotypes (166). Furthermore, knockdown of HDAC8 has been shown to inhibit cell proliferation in lung cancer, colorectal cancer, and cervical cancer cell lines, highlighting its potential as a therapeutic target across multiple cancers (167–169).

##### 2.2.1.2 Class II HDACs

Class II HDACs are divided into two subfamilies: Class IIa (HDAC4, 5, 7, 9) and Class IIb (HDAC6 and 10) (154). Some members of the Class IIa subfamily play dual roles in cancer. HDAC4, for example, is upregulated in breast cancer patients, yet its inhibition or downregulation can also affect cancer progression. Interestingly, homozygous deletion of HDAC4 has been observed in melanoma cell lines, indicating that HDAC4 can function both as an oncogenic factor and as a tumor suppressor depending on the context (170). Similarly, HDAC7 demonstrates dual roles in tumor biology. High expression of HDAC7 and HDAC9 has been linked to poor prognosis in children with acute lymphoblastic leukemia (ALL), whereas Skov et al. reported significant downregulation of HDAC7 in myeloproliferative tumors (171, 172). These findings suggest that Class IIa HDACs may serve as both proliferation-promoting and tumor-suppressive factors, depending on the cellular environment. In addition, Peixoto et al. reported that HDAC5 is essential for the replication fork process in cancer cells, as it is capable of maintaining and assembling the structure of heterochromatin around centromeres in cancer cells (173).

Among the Class IIb HDACs, HDAC6 has been extensively studied for its role in tumorigenesis. Elevated HDAC6 expression is positively correlated with cancer progression in oral squamous cell carcinoma, and estrogen stimulation increases HDAC6 gene expression in MCF-7 breast cancer cells (174). Moreover, acute myeloid leukemia samples and leukemia cell lines (such as HL60, K562, and KG1a) present elevated levels of HDAC6 expression (175). HDAC10, another member of Class IIb, has been implicated in gastric cancer development. It plays a crucial role in regulating the production of reactive oxygen species (ROS) in gastric cancer cells. Inhibition of HDAC10 leads to ROS accumulation, which triggers apoptosis in these cells, suggesting its potential as a therapeutic target (176).

##### 2.2.1.3 Class III HDACs

Sirtuins, Class III HDACs, play both oncogenic and tumor-suppressive roles in cancer, depending on the context. Various sirtuins have been found to be aberrantly expressed in multiple types of cancers. The most studied of these genes is SIRT1, which is widely recognized as a key epigenetic regulator involved in numerous biological processes (177). SIRT1 is responsible for deacetylating histone H1 lysine 26 (H1K26ac), histone H3 lysine 9 (H3K9ac), and histone H4 lysine 16 (H4K16ac) (178). Compared with that in normal tissues, the overexpression of SIRT1 has been reported in several cancers, including prostate cancer, colorectal cancer, leukemia, and melanoma. However, SIRT1 downregulation has also been observed in other types of tumors, such as breast cancer and hepatocellular carcinoma (179, 180), reflecting its multifaceted effects that depend on its subcellular localization and roles in different tissues.

On the other hand, SIRT2 primarily acts as a tumor suppressor. Studies have shown that a deficiency in SIRT2 impairs the mitotic checkpoint, leading to genomic instability and tumorigenesis (181). Furthermore, a significant decrease in SIRT2 expression has been reported in patients with gliomas (182). In cholangiocarcinoma, SIRT3 plays a tumor-suppressive role by downregulating the HIF1α/PDK1/PDHA1 pathway, leading to tumor regression (183). SIRT3 also inhibits renal cancer tumorigenesis by blocking mitochondrial biogenesis and inducing ferroptosis. In gallbladder cancer, SIRT3 suppresses tumors by inhibiting AKT-dependent mitochondrial metabolism and epithelial−mesenchymal transition (EMT) (184). However, in cervical cancer, SIRT3 overexpression promotes cancer cell progression by reprogramming fatty acid synthesis.

SIRT7, another member of this class, is a promoter-associated, highly selective H3K18Ac deacetylase. It plays a role in mediating transcriptional repression and stabilizing the cancer cell phenotype, further underscoring the diverse and complex roles of sirtuins in cancer.

##### 2.2.1.4 Class IV HDACs

HDAC11, a newly discovered member of the HDAC family, is encoded by a gene located on human chromosome 3q25.1 and is found in both the nucleus and cytoplasm (185). Its role in cancer appears to be cancer type specific. HDAC11 is highly expressed in lung adenocarcinoma and squamous cell carcinoma, where it is associated with poor patient prognosis (186, 187). In these cancers, HDAC11 promotes tumor cell migration, stemness, and drug resistance. In contrast, in basal-like breast cancer (BLBC), HDAC11 expression is downregulated, and overexpression of HDAC11 can suppress *in vitro* invasion and *in vivo* metastasis in xenograft breast cancer models, such as the SUM1315 and BT549 cell lines (128, 188).

The role of HDAC11 in hepatocellular carcinoma (HCC) metastasis is complex and may depend on the specific cancer cell lines and preclinical models used. Wang et al. reported that the downregulation of HDAC11 significantly reduced the migration and invasion abilities of highly metastatic MHCC97H HCC cells (189). However, Zhu et al. reported that while the knockdown of HDAC11 inhibited the proliferation of HepG2 cells, it did not affect invasion or migration (190).

In addition to its role in metastasis, HDAC11 is implicated in maintaining stemness and promoting tumor development in HCC. Downregulation of HDAC11 significantly suppresses glycolysis in HCC cancer stem cells and inhibits the stem cell-like properties of these cells by modulating their glycolytic levels (191). These findings highlight the context-dependent functions of HDAC11 across different cancer types.

### 2.3 Histone ubiquitination

All core histone proteins can undergo ubiquitination, but H2A and H2B are the most ubiquitinated and commonly modified histones in the nucleus (192, 193). Histone H2A was the first identified ubiquitination substrate, with lysine 119 (H2AK119ub) serving as the single ubiquitination site, catalyzed by the polycomb repressive complex 1 (PRC1) E3 ligase (194–196). H2AK119ub1 is highly enriched in the promoter regions of polycomb target genes and functions as a transcriptional repressor through various mechanisms. Histone H2A ubiquitination (H2Aub) plays a key role in several biological processes, including gene transcription and DNA damage repair (197, 198). Additionally, H2Aub promotes the binding of histone H1 to the nucleosome, stabilizing the nucleosome by preventing the dissociation of DNA from it (199).

Similarly, the ubiquitination site for histone H2B in mammals is typically located at lysine 120 (H2BK120ub) (200). *In vivo*, the ubiquitination of H2B (H2BK123ub) is catalyzed by the E2 transferase Rad6 and the E3 ligase Bre1. H2Bub rapidly accumulates at double-strand break (DSB) sites, where it plays a pivotal role in DSB repair (201, 202). Studies indicate that H2BK123ub promotes the methylation of histone H3 at lysines 4, 46, and 79 (H3K4, H3K46, H3K79) and that methylation at H3K46 and H3K79 is essential for effective DSB repair (203). These dynamic post-translational modifications regulate gene transcription and DNA repair through several mechanisms, including histone−DNA interaction regulation, nucleosome stability, histone eviction, chromatin compaction, histone cross-talk, and the recruitment of effector proteins.

In cancer, the mechanisms that regulate histone ubiquitination are frequently disrupted. Abnormal histone ubiquitination can drive tumorigenesis by altering the expression of tumor suppressors and oncogenes, misregulating cell differentiation, and promoting the proliferation of cancer cells. Compared with that in normal tissues, H2AK119ub1 expression is generally reduced in prostate cancer tissues. A global loss of H2BK120ub has been observed in patients with triple-negative breast cancer, gastric cancer, and colorectal cancer. Depletion of H2BK120ub significantly decreases p53 expression while simultaneously promoting c-MYC expression (204–207). Furthermore, DNA and RNA sequencing data revealed that genes encoding histone E3 ubiquitin ligases are frequently altered in cancer.

The polycomb E3 ubiquitin ligase subunits RING1A, RING1B, and BMI1, along with H2AK119ub1, help maintain the adult stem cell pool, and they may also contribute to the maintenance of cancer stem cells (208). In leukemia cells, BMI1 promotes cancer cell self-renewal by mediating the repression of key tumor suppressor genes (including the INK4A/ARF locus) through H2AK119ub1 (209). In addition to promoting acute leukemia, BMI1 promotes the proliferation of cancer cells in various solid tumors, including gastric cancer, pancreatic cancer, and epithelial ovarian cancer, by catalyzing H2A ubiquitination at lysine 119 (H2AK119ub) (210–212).

Given these roles, targeting abnormal histone ubiquitination represents a viable strategy for reprogramming transcription in cancer cells. The development of inhibitors that target aberrant histone ubiquitination sites or E3 ubiquitin ligases to block cancer cell proliferation and induce cell death is a promising avenue in histone ubiquitination-targeted cancer therapies.

### 2.4 Histone lactylation

Lactylation, a newly discovered post-translational modification (PTM) of histones, is closely related to the glycolytic metabolite lactate. Therefore, histone lactylation plays a significant role in cellular metabolic reprogramming. In 2019, Zhang et al. first reported a novel post-translational modification (PTM) of histones induced by lactate-lactylation (213). They discovered that lysine residues in histone tails can undergo lactylation. Subsequent studies confirmed the widespread existence of lactylation in various cancers and its close association with processes such as the development of malignancies. In hepatocellular carcinoma (HCC), researchers have reported that histone lactylation activates the transcription of ESM1 in HCC cells and that ESM1 is highly expressed in HCC, where it plays a carcinogenic role. Histone lactylation promotes the malignant phenotype of cells, tumor growth, and metastasis by increasing the expression of ESM1 in HCC. These findings may provide new therapeutic targets for the treatment of HCC (214). Yang et al. reported that the level of histone lactylation is associated with poor prognosis in patients with clear cell renal cell carcinoma (ccRCC). Mechanistic studies have shown that histone lactylation can promote the progression of ccRCC by activating the transcription of platelet-derived growth factor receptor β (PDGFRβ). Targeting histone lactylation can inhibit the proliferation and metastasis of ccRCC cells *in vivo* (215). In addition to the pro-cancer effects, histone lactylation can also have tumor-suppressive effects.

According to a study by Jiang et al. on non-small cell lung cancer (NSCLC), increased histone lactylation can lead to decreased levels of hexokinase 1 (HK-1) and pyruvate kinase M (PKM) in glycolysis, as well as increased levels of succinate dehydrogenase (SDHA) and isocitrate dehydrogenase 3γ (IDH3γ) in the tricarboxylic acid (TCA) cycle. This further results in the inhibition of tumor cell glycolysis, as well as reduced cell proliferation and migration abilities (216). In recent years, there have been several advancements in research on the roles of lactylation-related enzymes in tumors. Jin et al. reported that the delactylase SIRT3 can suppress the proliferation of liver cancer cells by regulating the lactylation of Cyclin E2 (217). In future research, studying histone lactylation modifications and their regulatory sites may lead to the identification of effective therapeutic targets for cancer treatment.

## 3 Clinical therapeutics

The previous discussion highlighted numerous abnormal histone modification sites and alterations in the activity of histone-modifying enzymes in cancer. These aberrant modifications and changes in the expression of modifying enzymes can serve as valuable biomarkers for the accurate screening, detection, diagnosis, and prognosis of tumors. Since different enzymes catalyze modifications at specific histone sites, targeting these histone-modifying enzymes with oncogenic potential represents a promising strategy in cancer therapy.

### 3.1 Epigenetic markers for prognosis

Epigenetic changes, particularly histone modifications observed in the early stages of tumor development and cancer progression, have been proposed as biomarkers for early cancer detection, prognosis, and treatment response. In most breast cancer patients, low or absent expression of H4K16ac has been identified, making H4K16ac an early marker of the disease (218). During epithelial−mesenchymal transition (EMT), the loss of H4K16ac in mesenchymal cells can distinguish between epithelial and mesenchymal phenotypes. Additionally, low levels of H4R3me2, H3K4me2, and H4K20me3 are associated with poor prognosis in patients with breast cancer (219). In gastric cancer, Jang et al. reported high expression of H3K9me3, which is positively correlated with tumor stage. Abnormal expression of KDM5B also promotes gastric cancer metastasis by regulating various signaling pathways, contributing to poor prognosis. In ovarian cancer, overexpression of HDAC3 and loss of H3K27me3 are linked to prognosis and disease progression. Furthermore, in glioblastoma (GBM), the nuclear expression levels of lysine methyltransferases, such as SETDB1, KMT5B, Suv-39h1, and EZH2, are elevated and associated with advanced histological cancer grades. Overall, changes in histone post-translational modifications have demonstrated clinical utility and are increasingly recognized as promising biomarkers for early cancer detection and diagnosis.

### 3.2 New targets and therapy

#### 3.2.1 HMT inhibitors

Increasing evidence suggests that histone methyltransferases (HMTs) may serve as potential therapeutic targets for cancer treatment. As a result, the development of HMT inhibitors has gained considerable attention over the past decade. Most of these inhibitors target enzymes such as EZH2 and PRMT5 (220). To date, the U.S. FDA has approved two small-molecule inhibitors: tazemetostat (EPZ-6438) and valemetostat (DS-3201b), which target EZH2 or both EZH1 and EZH2 for cancer therapy. In addition, multiple EZH2 inhibitors (EZH2is), such as CPI-1205, SHR2554, and PF-06821497, are currently in various stages of clinical trials. PRMT5, another promising therapeutic target, is also being explored for cancer treatment. GSK3326595, a PRMT5 inhibitor, is in phase I trials for non-Hodgkin lymphoma, while other PRMT5 inhibitors, such as the PRT543 series, are in phase I trials for relapsed/refractory advanced solid tumors, and the PRT811 series is in phase I trials for relapsed glioma, advanced solid tumors, and CNS lymphoma. Other HMT inhibitors, such as the DOT1L inhibitor EPZ-5676, which is undergoing clinical trials for AML, ALL, and CML, have made significant progress in recent years.

While substantial progress has been made in the development of HMT inhibitors, the emergence of resistance with prolonged use highlights the limitations of monotherapy in treating solid tumors. Studies have shown that combining HMT inhibitors, such as EZH2 inhibitors, with immunotherapy, targeted therapy, chemotherapy, and endocrine therapy can result in synergistic anti-tumor effects, improving treatment outcomes. However, owing to the high homology between EZH1 and EZH2, EZH2 inhibitors often inhibit both, presenting challenges for the development of highly specific EZH2 inhibitors. Adachi et al. reported the discovery of two novel orally bioavailable dual-target EZH1/2 inhibitors, OR-S1 (compound 1) and OR-S2 (compound 2). These compounds exhibit potent and selective inhibition of both EZH2 and EZH1, effectively reducing H3K27me3 levels, and have demonstrated significant anti-tumor activity against diffuse large B-cell lymphoma cells with gain-of-function mutations. Optimizing the therapeutic efficacy of these inhibitors and minimizing their toxicity and side effects will be the focus of future research.

Inhibition of EZH2 can boost tumor immunotherapy through various mechanisms. Resistance to immune checkpoint inhibitors (ICIs) is currently a significant challenge in cancer immunotherapy. DuCote et al. reported that EZH2 inhibitors could increase ICI responses in patients undergoing treatment for lung squamous cell carcinoma (LSCC) (221). In LSCC, tumors can evade the immune system through the expression of PD-L1, thereby suppressing T-cell activation. Additionally, these tumors secrete high levels of CXCL1/2/3, which recruit T-cell-suppressive neutrophils and express high levels of arginase, further driving T-cell suppression. Under EZH2 inhibition, tumors upregulate the antigen presentation mechanisms of MHC I and MHC II and shift from the expression of CXCL1/2/3 to the expression of the T-cell-promoting cytokines CXCL9/10/11 and the anti-inflammatory molecule ALOX15. The combined use of anti-PD1 therapy and EZH2 inhibitors (such as GSK126 or EPZ6438) has also shown significant tumor-suppressive effects in LSCC patients. Furthermore, the EZH2 inhibitor tazemetostat has recently received FDA approval and is currently in clinical trials for the use of ICI therapy in urothelial carcinoma (NCT03854474). These studies suggest that EZH2 inhibition combined with ICI treatment may be an effective treatment strategy for ICI-resistant solid tumors.

#### 3.2.2 HDM inhibitors

The targeting of demethylases is an emerging approach for the treatment of various cancers, and numerous demethylase inhibitors have been reported, some of which are currently undergoing clinical evaluation for cancer therapy. KDM inhibitors can be classified into two categories: KDM1 and JmjC family histone lysine demethylase inhibitors. Trans-2-phenylcyclopropylamine (TCP) and its derivatives can inhibit KDM1A and KDM1B (84, 222). Currently, six TCP-based KDM1A inhibitors have been developed and are in clinical trials, including TCP, ORY-1001, ORY-2001, GSK-2879552, INCB059872, and IMG-7289, which covalently bind to the FAD domain within KDM1A (84, 223). ORY-1001 inhibits the proliferation of TNBC cells and induces apoptosis by inactivating AR (224). The TCP derivatives (NCL-1, NCD-38, MC_2580, and DDP_38003) also exhibit anti-tumor effects as KDM1 inhibitors. NCL-1 and NCD-38 inhibit KDM1A, leading to reduced viability and increased apoptosis of GSCs, with minimal effects on differentiated cells (90) Maes et al. emphasized the therapeutic potential of combining ORY-1001 with checkpoint inhibitors for the treatment of melanoma. After cotreatment with ORY-1001 and anti-PD1 antibodies, significant tumor growth inhibition (TGI) was achieved, which was 54% higher than that in the anti-PD1 antibody treatment group (225).

JmjC family inhibitors are divided into broad-spectrum and subfamily-specific inhibitors. IOX1 is a broad-spectrum inhibitor that targets several subfamilies, including KDM2, KDM3, KDM4, KDM5, KDM6, and KDM7 (226). Liu et al. reported that IOX1 inhibits the expression of P-glycoprotein (P-gp) in cancer cells through the JMJD1A/β-catenin/P-gp pathway, thereby reducing the expression level of PD-L1. Moreover, IOX1 significantly enhances immunogenic cell death (ICD) induced by doxorubicin (DOX), promotes T-cell infiltration, and markedly suppresses tumor growth in preclinical models. These results suggest that the combination of IOX1 with immune checkpoint inhibitors may represent a promising strategy for cancer treatment (227). On the other hand, SD49-7 is a small-molecule inhibitor specific to KDM4A and KDM4C. Inhibiting these demethylases disrupts the expression of MDM2, activates p21, and suppresses the stemness of leukemia cells, leading to increased cell apoptosis (228). PBIT is a potent, selective inhibitor of KDM5B that reduces the expression of JARID1B, which results in decreased levels of cancer stem cell (CSC) and epithelial-mesenchymal transition (EMT) markers in cisplatin-resistant non-small cell lung cancer (229). PBIT also enhances the sensitivity of cancer cells to radiation therapy. These small-molecule KDM inhibitors, which exhibit drug-like specificity and selectivity, can be used either as standalone therapies or in combination with other immunotherapies and chromatin-targeting agents, thereby offering more viable treatment options for cancer.

#### 3.2.3 HAT inhibitors

Among the existing small-molecule histone acetyltransferase (HAT) inhibitors, many studies have focused on compounds that target p300/CBP, which play key roles in acetylating histone H3 at lysines 18 and 27 (H3K18, H3K27) to facilitate gene activation critical for cell growth and differentiation (230). In addition to the enzymatic HAT domain, p300/CBP contains several other functional domains, such as three cysteine-histidine-rich domains (CH1, CH2, and CH3), a KIX domain, a bromodomain, and a steroid receptor coactivator interaction domain (SRC-1 interaction domain). Inhibitors targeting p300/CBP are designed primarily to exploit these domain characteristics.

One promising inhibitor is CCS1477, a CBP/p300 bromodomain inhibitor developed by CellCentric, which is currently in phase 1b/2a clinical trials for treating hematological malignancies and late-stage castration-resistant prostate cancer. A-485 is another potent and highly selective p300/CBP inhibitor that has shown anti-tumor activity in prostate cancer cell lines (231). Additionally, Ding et al. identified compound 13f, a novel p300/CBP HAT inhibitor, which demonstrated significant anti-tumor effects in an ovarian cancer xenograft mouse model (232).

Natural compounds have also shown promise. Garcinol, a natural inhibitor of p300/CBP and PCAF, has anti-tumor effects on several cancer cell lines, including hepatocellular carcinoma, gastric cancer, and triple-negative breast cancer (233). WM-3835 selectively inhibits HBO1, another member of the HAT family, and effectively suppresses mouse osteosarcoma (OS) tumor cell growth (234). Additionally, the small molecule NBP targets KAT7 to inhibit PD-L1 expression and weaken the PD-1/PD-L1 axis, reducing T-cell apoptosis to alleviate lung cancer progression. It may serve as a potential therapeutic strategy for immunotherapy in lung cancer (235).

Despite these advances, the development of targeted drugs for HAT inhibition remains in its early stages. Future research should focus on identifying and developing inhibitors for other members of the HAT family, offering new and effective strategies for anti-tumor therapy.

#### 3.2.4 HDAC inhibitors

Histone deacetylase (HDAC) inhibitors play crucial roles in restoring the balance of acetylation and deacetylation of lysine residues of histones and nonhistone proteins and are used to treat several diseases, including cancer (**Table 3**). Four HDAC inhibitors (HDACis), vorinostat (SAHA), belinostat (PXD101), panobinostat (LBH589), and romidepsin (FK228), have been approved by the US FDA for the treatment of cutaneous T-cell lymphoma (CTCL), relapsed or refractory peripheral T-cell lymphoma (PTCL), and multiple myeloma (MM), whereas chidamide (CS055) has also been approved by China’s NMPA for the treatment of relapsed or refractory peripheral T-cell lymphoma (249). Additionally, several HDAC inhibitors are in clinical trial stages.

**Table 3:** HDACis in cancer therapy.

HDAC inhibitors	HDAC type	Target	Cancer
Entinostat(MS-275)	Class I	HDAC1/HDAC3 /HDAC8	Breast cancer (236) Non-small cell lung cancer (166)
Tacedinaline (CI994)	Class I	HDAC1/HDAC3	Pancreatic cancer (237) Non-small cell lung cancer (238)
Mocetinostat (MGCD0103)	Class I	HDAC1/HDAC2/ HDAC3/HDAC11	Acute myeloid leukemia (239) Bladder cancer (240)
CXD101	Class I	HDAC1/HDAC2/ HDAC3	Colorectal cancer (241)
Droxinostat	Class I	HDAC3/HDAC6/ HDAC8	Prostate cancer (242)
Ricolinostat (ACY-1215)	Class II	HDAC6	Colorectal cancer (243) Multiple myeloma (244)
WT161	Class II	HDAC6	Multiple myeloma (245)
Tasquinimod	Class II	HDAC4	Prostate cancer (246)
LMK-235	Class II	HDAC4	Breast cancer (247)
NQN-1	Class II	HDAC6	Acute myeloid leukemia (248)

The majority of HDACi structures contain three pharmacophoric elements: a cap structure (Cap region) that interacts with amino acid residues at the entrance edge of the HDAC active site; a zinc-binding group (ZBG) that chelates the catalytic zinc ion located at the bottom of the active site pocket; and a linker region that connects the Cap region and ZBG, which interacts with the hydrophobic channel of the active site (250–252). The chemical structures of HDAC inhibitors can be divided into four main categories: hydroxamic acids (such as vorinostat), benzamide derivatives (such as chidamide), cyclic peptides (such as romidepsin), and fatty acids (such as 2-propylpentanoic acid).

Mocetinostat (MGCD0103) is a novel benzamide-class HDAC inhibitor that can inhibit HDAC1, 2, 3, and 11. It is currently being researched for diseases such as acute myeloid leukemia, bladder cancer, and non-small cell lung cancer and has entered phase II clinical trials (253). Largazole is a cyclic depsipeptide natural product that shows good activity and selectivity for HDAC1. It inhibits the proliferation of the human breast cancer cell line MDA-MB-231 and the human osteosarcoma cell line U2OS but has no effect on normal cells (254). AR-42 is an orally effective HDAC inhibitor that is currently in clinical trials for the treatment of multiple myeloma, leukemia, and lymphoma (255). CAY10603 is a potent HDAC6 inhibitor that effectively inhibits the activity of HDAC6. It inhibits the proliferation of Burkitt’s lymphoma cell lines and induces caspase-dependent apoptosis (256).

The combination of HDACis and immunotherapy has made great progress in cancer immunotherapy. The activation of the immune response by HDACis could prevent cancer relapse. Entinostat (ENT; Class I HDAC inhibitor) has shown significant efficacy in tumor immunotherapy when combined with anti-PD-1/anti-CTLA-4 antibodies, functioning by reducing the number of tumor-infiltrating G-MDSCs (257). Owing to the typical downregulation of MHC class I expression caused by epigenetic mechanisms in cancer, HDAC inhibitors can upregulate MHC class I expression in various types of cancer. Romidepsin (HDAC1/2 inhibitor), valproic acid (Class I HDAC inhibitor), or RGFP966 (HDAC3 inhibitor) can upregulate MHC class I expression and the expression of costimulatory molecules such as CD80 and CD86 in B-cell lymphomas (258). In HER2+ breast cancer, the combination of palbociclib and trastuzumab (anti-HER2) achieves anti-tumor efficacy by stimulating the release of CXCR3-reactive chemokines and increasing the recruitment of tumor-associated natural killer (NK) cells (259). In addition, HDAC inhibitors (HDACis) can modulate the effector functions of activated immune cells and potentiate the efficacy of immunotherapeutic strategies against established solid tumors. The HDAC inhibitor MS-275 can enhance the lymphocyte depletion induced by oncolytic virus vectors, leading to the selective exhaustion of conventional lymphocytes and regulatory T cells (Tregs) while allowing the expansion of antigen-specific secondary responses (260). HDAC inhibitors may participate in the anticancer immune response by directly altering the immunogenicity of tumor cells and potentially rescuing the functional activity of exhausted CD8+ T cells (261).

Currently, the main applications of HDAC inhibitors (HDACis) are limited to the treatment of peripheral T-cell lymphoma and cutaneous T-cell lymphoma, whereas the development of drugs for solid tumors is relatively limited. Additionally, clinical trials of HDAC inhibitors have revealed numerous adverse reactions in patients, including thrombocytopenia-induced bleeding, neutropenia-induced susceptibility to infections, anemia due to hemoglobin reduction, arrhythmias, myocardial hypertrophy, and neurotoxicity, which also pose major limitations in the development of HDAC inhibitors (262). Furthermore, most HDACis are broad-spectrum inhibitors, making the development of efficient inhibitors with cell selectivity and isoform specificity a significant challenge.

## 4 Summary and future perspectives

Epigenetic modifications have become key regulatory factors and drivers in the occurrence and development of cancer in recent years. The processes of DNA repair, replication, transcription, translation, and posttranscriptional and post-translational regulation are all under the control of epigenetics. Therefore, abnormal expression patterns or epigenomic alterations can lead to dysregulation, ultimately resulting in cancer. Changes in the epigenetic landscape of cancer can affect the expression of genes involved in cellular metabolism, primarily through the dysregulation of metabolic signaling pathways caused by abnormal DNA methylation, histone modifications, and non-coding RNAs.

Histone post-translational modifications (PTMs) do not alter the DNA sequence but can change the expression and functional levels, providing new explanations for many biological activities. In addition to common modifications such as methylation, acetylation, ubiquitination, and phosphorylation, in recent years, histone butyrylation, lactylation, propionylation, and isonicotinamide have gradually become new research hotspots. Most PTMs are reversible and therefore can regulate the functionality of the proteome in a cell type-specific manner to modulate gene expression. Abnormalities in histone post-translational modifications often affect key molecular regulatory mechanisms involved in the progression of tumors.

In this review, we elaborate on the main types of histone PTMs and their functions in different categories of cancers. We subsequently summarize the key enzymes that influence histone modifications and their inhibitors, providing feasible targets for cancer treatment research. Furthermore, the crosstalk between different histone PTMs is also an interesting research direction. Methylation of H3K4 can increase the acetylation activity of HATs on the H3 tail. Therefore, the impact of histone modification enzymes on cancer development is certainly not singular.

As the role of histone PTMs in the cancer field has gained increasing attention, targeted drugs against histone modification enzymes have also become a research focus in this area. As mentioned previously, HDAC inhibitors (HDACis) are currently the most widely used HDACis in clinical practice. While various HDAC inhibitors that target different HDAC classes have been approved, these inhibitors are currently limited to the treatment of hematological cancers, with few HDAC inhibitors that target solid tumors. Therefore, developing more HDAC inhibitors that target solid tumors is also a future research focus for researchers. Furthermore, since the therapeutic efficacy of HDAC inhibitors as single agents is limited, there is a need to develop dual-target HDAC drugs. Dual-target HDAC inhibitors can act on multiple signaling pathways involved in tumor development, thereby more comprehensively regulating epigenetic modifications and gene expression in cells. For example, researchers at China Pharmaceutical University have recently developed a series of innovative dual PD-L1/HDAC6 inhibitors, C1-C6 (patent number: CN113387840A). In the CT26 syngeneic colon tumor model, the tumor inhibition effect of C5 exceeded the efficacy of BMS202 (a PD-L1 inhibitor) or SAHA (an approved HDAC inhibitor) monotherapy.

Histone modification enzyme inhibitors are also a hot topic in the clinical treatment of cancer immunotherapy. Immunotherapy works by activating the immune system to fight cancer cells, achieving good efficacy in some patients. However, in many patients, tumor cells evade detection by the immune system because of their low mutational burden, resulting in “cold tumors.” Additionally, cancer cells can develop various resistance mechanisms to immune checkpoint inhibitors, such as reduced antigen presentation, modulation of immune cell recruitment, secretion of immunosuppressive factors, decreased co-stimulatory molecules, and induction of T cell apoptosis. These factors greatly limit the effectiveness of immunotherapy in cancer patients. Currently, multiple studies have shown that histone modification enzyme inhibitors, important epigenetic modifiers, can not only enhance anti-tumor immunity by inhibiting the expression of immune checkpoint molecules (ICMs) but also be used in combination with immune checkpoint inhibitors (ICIs) to enhance the tumor response to immunotherapy by increasing ICM expression. A study revealed that the combination of HDAC inhibitors (HDACis) and immune checkpoint inhibitors (ICIs) altered the infiltration and function of innate immune cells, leading to a more robust adaptive immune response via the inhibition of myeloid-derived suppressor cells (MDSCs) and immune-resistant breast tumors (263). Many drugs targeting dysregulated epigenetic regulators have entered clinical use for the treatment of hematological malignancies. Increasing compelling evidence suggests that epigenetic therapies have the potential to transform immunosuppressive (“cold”) tumors into immunopermissive (“hot”) tumors. The combination of epigenetic therapies and immunotherapy can consolidate anti-tumor immune responses, reprogram the immunosuppressive TME, and improve treatment outcomes.

In future cancer treatment, the ability of HDAC to synergize with other therapies makes it a promising candidate for cancer immunotherapy. In future tumor treatment strategies centered on histone PTMs, only by clearly elucidating the specific mechanisms of action of histone PTMs in tumors will it be possible to conduct research on targeted anticancer drugs based on the molecular mechanisms of PTMs and thus promote cancer treatment.
